# *Candidatus Bartonella merieuxii*, a Potential New Zoonotic *Bartonella* Species in Canids from Iraq

**DOI:** 10.1371/journal.pntd.0001843

**Published:** 2012-09-27

**Authors:** Bruno B. Chomel, Audrey C. McMillan-Cole, Rickie W. Kasten, Matthew J. Stuckey, Shingo Sato, Soichi Maruyama, Pedro P. V. P. Diniz, Edward B. Breitschwerdt

**Affiliations:** 1 Department of Population Health and Reproduction, School of Veterinary Medicine, University of California Davis, Davis, California, United States of America; 2 Public Health Command Region-Pacific, Tripler Army Medical Center, Honolulu, Hawaii, United States of America; 3 Laboratory of Veterinary Public Health, Department of Veterinary Medicine, College of Bioresource Sciences, Nihon University, Fujisawa, Japan; 4 College of Veterinary Medicine, Western University of Health Sciences, Pomona, California, United States of America; 5 Intracellular Pathogens Research Laboratory, Center for Comparative Medicine and Translational Research, College of Veterinary Medicine, North Carolina State University, Raleigh, North Carolina, United States of America; University of California San Diego School of Medicine, United States of America

## Abstract

Bartonellae are emerging vector-borne pathogens infecting erythrocytes and endothelial cells of various domestic and wild mammals. Blood samples were collected from domestic and wild canids in Iraq under the United States Army zoonotic disease surveillance program. Serology was performed using an indirect immunofluorescent antibody test for *B. henselae*, *B. clarridgeiae*, *B. vinsonii* subsp. *berkhoffii* and *B. bovis*. Overall seroprevalence was 47.4% in dogs (n = 97), 40.4% in jackals (n = 57) and 12.8% in red foxes (n = 39). *Bartonella* species DNA was amplified from whole blood and representative strains were sequenced. DNA of a new *Bartonella* species similar to but distinct from *B. bovis*, was amplified from 37.1% of the dogs and 12.3% of the jackals. *B. vinsonii* subsp. *berkhoffii* was also amplified from one jackal and no *Bartonella* DNA was amplified from foxes. Adjusting for age, the odds of dogs being *Bartonella* PCR positive were 11.94 times higher than for wild canids (95% CI: 4.55–31.35), suggesting their role as reservoir for this new *Bartonella* species. This study reports on the prevalence of *Bartonella* species in domestic and wild canids of Iraq and provides the first detection of *Bartonella* in jackals. We propose *Candidatus* Bartonella merieuxii for this new *Bartonella* species. Most of the *Bartonella* species identified in sick dogs are also pathogenic for humans. Therefore, seroprevalence in Iraqi dog owners and bacteremia in Iraqi people with unexplained fever or culture negative endocarditis requires further investigation as well as in United States military personnel who were stationed in Iraq. Finally, it will also be essential to test any dog brought back from Iraq to the USA for presence of *Bartonella* bacteremia to prevent any accidental introduction of a new *Bartonella* species to the New World.

## Author Summary

Bartonellae are emerging vector-borne pathogens infecting erythrocytes and endothelial cells of various domestic and wild mammals. Blood samples were collected from domestic and wild canids in Iraq under the United States Army zoonotic disease surveillance program. Serology was performed using an indirect immunofluorescent antibody test for *B. henselae*, *B. clarridgeiae*, *B. vinsonii* subsp. *berkhoffii* and *B. bovis*. Overall seroprevalence was 47% in dogs (n = 97), 40% in jackals (n = 57) and 13% in red foxes (n = 39). *Bartonella* species DNA was amplified from whole blood and representative strains were sequenced. DNA of *Candidatus* Bartonella merieuxii, a new *Bartonella* species similar to but distinct from *B. bovis*, was amplified from 37% of the dogs and 12% of the jackals. *B. vinsonii* subsp. *berkhoffii* was also amplified from one jackal and no *Bartonella* DNA was amplified from foxes. Dogs were more likely to be *Bartonella* PCR positive than wild jackals and foxes, suggesting the role of dogs as reservoir for this new *Bartonella* species. As most *Bartonella* species isolated or detected in dogs are also infecting humans, it will be important to test Iraqi people, especially from Baghdad, and American veterans who served in Iraq for the presence of infection by Candidatus Bartonella merieuxii.

## Introduction

*Bartonella* are fastidious, hemotropic, gram negative, rods that have an affinity for the erythrocytes, endothelial cells and macrophages of their hosts [1]–[3]. These bacteria cause vasoproliferative lesions by entering endothelial cells resulting in cellular proliferation and migration [4]. The organisms are highly adaptive which enables them to persist within the cells of their mammalian hosts for extended periods [2]. Culturing the bacteria is often frustrating [5], [6], as colonies can take up to 45 days to grow on enriched blood media [2].

These bacteria are primarily arthropod-borne [1]–[3]; the main vectors definitively identified are the sand fly (*Lutzomyia verrucarum*) for *B. bacilliformis*, the cat flea (*Ctenocephalides felis*) for *B. henselae*, and the human body louse (*Pediculus humanis corporis*) for *B. quintana* [5]. Vector-borne transmission of *Bartonella* to bank voles and gerbils (*Gerbillus nanus*, *G. dasyurus*) by *Ctenophthalmus nobilis* fleas and to jirds (*Meriones crassus*) by *Xenopsylla ramesis* has also been established [7], [8]. *Bartonella* DNA has been detected in ticks and biting flies as well [9], [10], suggesting many more possible arthropod vectors [2], [3], recently supported through experimental demonstration of vector competence for *B. birtlesii* by *Ixodes ricinus* [11].

More than 25 species/subspecies of *Bartonella* and many Candidatus species have been identified in a wide range of domestic and wild animals [10; http://www.bacterio.cict.fr]. Of these, 14 are recognized as zoonotic or potentially zoonotic, including 10 isolated from dogs and cats.

The clinical spectrum of *Bartonella* infections in humans is quite broad [1], [3], [12]. Worldwide, cat scratch disease (CSD) is the most commonly recognized zoonotic disease caused by *Bartonella* species [2] that occurs in immunocompetent individuals [1]–[3]. *B. henselae* and *B. quintana* cause severe systemic diseases in immunocompromised patients, such as bacillary angiomatosis and bacillary peliosis [1]–[3], [12]. *Bartonella* infections are an important cause of culture-negative endocarditis in humans [13], comprising an estimated 3–10% of all endocarditis cases [13], [14]. Other manifestations of *Bartonella* infections include prolonged fever of unknown origin with or without lymphadenopathy [15]. When using an insect based enrichment medium, people with extensive arthropod exposure and animal contact had high infection rates (24–41%) of *Bartonella* spp. [16].

Several *Bartonella* species, most of which are zoonotic, have been isolated or detected by PCR amplification and DNA sequencing from dogs, including *B. vinsonii* subsp. *berkhoffii*, *B. henselae*, *B. clarridgeiae*, *B. rochalimae*, *B. quintana*, *B. koehlerae*, *B. elizabethae*, *B. vinsonii* subsp. *arupensis*, *B. taylorii*, *B. volans*-like, *B. bovis* and *B. bovis*-like [3], [17]–[20]. Recently, six Italian dogs and one Greek dog were infected with a novel, uncultured *Bartonella* sp. strain designated as strain HMD [GenBank accession number EF614393], which is phylogenetically similar, but distinct from *B. bovis* [19]. As the abnormalities observed in dogs infected with *Bartonella* spp. are very similar to the clinical and pathologic abnormalities seen in humans, the findings in this manuscript may prove to be of comparative medical importance [2], [3]. The role of dogs as a primary reservoir for various *Bartonella* species is still unclear [2]. Nevertheless, dogs are excellent sentinels as part of a surveillance system for the detection of human infections [21]. In Iraq, limited information is available on zoonotic diseases in canids. A recent study investigated the presence of Q fever antibodies in military working dogs (MWD) deployed in Iraq and free-roaming dogs eliminated through a feral animal control program in locations throughout Iraq [22]. None of the MWD seroconverted versus 5.5% of the indigenous dogs. To our knowledge, presence of *Bartonella* infection has never been reported in Iraq. Therefore, the objective of the present study was to investigate the presence of *Bartonella* spp. by PCR amplification and to assess seroprevalence of *B. henselae*, *B. clarridgeiae*, *B. vinsonii* subsp. *berkhoffii* and *B. bovis* in domestic and wild canids of Iraq.

## Materials and Methods

### Subject enrollment, sample collection, and storage

Whole blood samples (1.5–2 ml) were collected between February and December 2008 under the US Army feral animal control and zoonotic disease surveillance program from the saphenous or jugular veins in EDTA tubes from 193 canids, including 97 (50.3%) stray dogs (*Canis familiaris*), 57 (29.5%) jackals (*Canis aureus*) and 39 (20.2%) red foxes (*Vulpes vulpes*) located on several United States military bases throughout Iraq. Categorical age, sex, and location data was also collected with samples, when possible.

### Ethics statement

All animals were chemically restrained prior to blood collection and humanely treated prior to euthanasia, in accordance with the rules of the ethic committee from the US Army feral animal control and zoonotic disease surveillance program and by the University of California, Davis (UCD) Animal Use and Care Committee (Protocol # 12668 originally approved on March 8, 2007) in accordance with the Guide for the Care and Use of Laboratory Animals of the National Institutes of Health. The blood samples were frozen and shipped to the UCD School of Veterinary Medicine. In a pilot study on 40 dogs, 10 jackals and 35 foxes ((McMillan-Cole, Master of Preventive Veterinary Medicine, Davis, CA; data not shown), either major bacterial contamination or lack of isolation of *Bartonella* was observed when blood culture were attempted. Therefore, the blood samples analyzed in the present study were only processed by DNA extraction.

### Serological analysis

Presence of antibodies against *B. henselae* H1 (ATCC 49882) and U4 (UC Davis), *B. clarridgeiae* (ATCC51734), *B. vinsonii* subsp. *berkhoffii* genotype I (ATCC 51672) and *B. bovis* (strain 91-4T: CIP 106692T and CCUG 43828T) were tested on the serum or blood supernatant samples, using an indirect immune-fluorescent-antibody assay (IFA) as previously described [18], [23], [24]. Serum samples were diluted to 1∶32 and 1∶64 and 20 µl of each dilution was added to the test wells. Positive and negative controls were included on each slide. Three conjugate (Cappel fluorescein-conjugate goat anti-dog IgG fraction, MP Biomedicals, Aurora, OH, USA) dilutions were used for the four antigens being tested: 1∶1400 (*B. vinsonii* subsp. *berkhoffii* and *B. bovis*), 1∶2800 (*B. henselae H1* and *U4*) and 1∶3600 (*B. clarridgeiae*). For each well, 20 µl of the conjugates were added to the respective slides. The intensity of the fluorescence was graded subjectively by two independent masked readers, with sample fluorescence graded ≥2 at the 1∶64 dilution reported as positive.

### DNA extraction and PCR-RFPL

The DNA was extracted as previously described [25]. Polymerase chain reaction-restriction fragment length polymorphism (PCR-RFLP) analysis of the *gltA* gene was then performed for all samples [25]. The primers used for the *gltA* gene were BhCS.781p 5′- and BhCS.1137n 5′, as previously described [26]. An approximately 400 bp fragment of the *gltA* gene was amplified and then verified by gel electrophoresis. The amplified product was then digested with restriction endonucleases *TaqI* (Promega, Madison, WI), *Hha*I, *Mse*I and *Dde*I (New England BioLabs, Ipswich, MA, USA). Banding patterns were compared with 19 standard strains of *Bartonella*, including *B. henselae* and *B. clarridgeiae*, *B. koehlerae* and *Bartonella* ruminant strains.

### Sequencing and phylogenetic analysis

Two representative DNA products from the dog and jackal groups were further analyzed by PCR of the 16S-23S rRNA intergenic spacer region (ITS) [27] and of the *rpoB* gene [28] and submitted to sequencing. As the *gltA* gene sequence for the HMD strain had not been previously performed, DNA from strain HMD was submitted by one of the authors (EB), and after PCR testing with the appropriate primers, was submitted for sequencing (Davis Sequencing, Davis, CA, USA). Amplified products from the *gltA* gene, *rpoB* gene, and the ITS region were sequenced in both directions and consensus sequences were compared. The sequence alignments obtained in this study were also referenced against those of other known *Bartonella* species deposited in the GenBank/EMBL/DDBJ database using BLAST [Table 1]. The Clustal W program [29] was used to align each isolate and compare homologous *gltA*, *rpoB* and ITS spacer sequences to identify genetic variants. A phylogenetic tree was constructed from concatenated sequences of *gltA*, *rpoB* and ITS from representative *Bartonella* isolates, including ruminant-associated *Bartonella* species, by using neighbor-joining methods. Nucleotide substitution rates were calculated by Kimura's two-parameter distance model, as previously described [30], using MEGA version 5.0 software [31]. Bootstrap analysis was performed with 1,000 trials.

**Table 1:** GenBank sequences used for Figure 1.

*Bartonella* species	*glt*A	*rpo*B	ITS
Iraq Jackal F040	This study	This study	This study
Iraq Dog F059	This study	This study	This study
*Bartonella* sp. HMD	JX073031	EF592104	EF614393
*B. bovis*	AF293394	AY166581	AY116638
*B. capreoli*	AF293392	AB290188	AB498009
*B. schoenbuchensis*	AJ278183	AY167409	AY116639
*B. chomelii*	AY254308	AB290189	AB498010
*B. elizabethae*	Z70009	AF165992	L35103
*B. henselae*	L38987	AF171070	L35101
*B. koehlerae*	AF176091	AY166580	AF312490
*B. quintana*	Z70014	AF165994	L35100
*B. vinsonii arupensis*	AF214557	AY166582	AF312504
*B. vinsonii berkhoffii*	U28075	AF165989	AF167988
*B. vinsonii vinsonii*	Z70015	AF165997	L35102
*B. rudakovii*	EF682090	EF682088	EF682087
*B. rochalimae*	DQ683195	DQ683198	DQ683199
*B. clarridgeiae*	U84386	AF165990	AF312497
*B. bacilliformis*	AB292601	AF165988	L26364

### Statistical analysis

*Bartonella* prevalence data generated from diagnostic tests and binomial proportion confidence intervals (CI) were calculated using Wald and Fisher's exact methods where appropriate in OpenEpi, version 2.3.1 (Dean AG, Sullivan KM, Soe MM. OpenEpi: Open Source Epidemiologic Statistics for Public Health). All variables were initially screened using univariate logistic regression to check for statistically significant associations with serology and PCR outcomes. Due to quasi-complete separation of PCR data, species and location variables were reclassified in biologically meaningful groups prior to univariate analysis. Using forward stepwise selection methods, categorical variables were retained in separate multivariate logistic regression models for PCR and IFA. Variables that induced changes >10% in the β-coefficients of covariates after inclusion in the model were considered to be potential confounders, and evaluated along with biological reasoning and prior knowledge. Likelihood ratio tests comparing models with and without two-way interaction terms were used to determine presence of effect measure modification. All logistic regression models and resulting adjusted odds ratios with 95% CI were generated in SAS, version 9.3 (SAS Institute Inc., Cary, NC).

## Results

### Dogs

Most of the dogs (79.38%; 77/97) were captured in Baghdad and surrounding suburbs (Inner Baghdad: 35, North Baghdad: 27, Outer Baghdad: 15). The other dogs were from locations classified as North Iraq (n = 15), West Iraq (n = 3) or South Iraq (n = 2). Forty-three (57.3%) of the 75 dogs for which sex was reported (missing from 22 dogs) were male. Age was not recorded for 27 animals, with 60 (85.7%) of the 70 dogs classified as adults. Thirty-six dogs (37.1%; 95% CI = 27.5%–46.73%) were PCR positive [Table 2]. RFLP analysis of the *gltA* PCR product from the four enzyme digestions indicated similar banding patterns for all 36 dogs (data not shown). Banding pattern was similar to *B. clarridgeiae* using *Taq*I, *Hha*I and *Mse*I. However, *Dde*I allowed differentiating these strains from *B. clarridgeiae*. A representative dog “strain” (F059) was chosen for further genetic analysis. Sequencing of the *gltA* gene fragment showed that the closest related *Bartonella* species were respectively *B. bovis*, *B. schoenbuchensis*, *B. melophagi*, *B. chomelii* and *B. capreoli* [Table 3]. The partial *gltA* sequence of the HMD strain was deposited in GenBank under the number JX073031. Sequencing of the 16S-23S ITS spacer region indicated that DNA of “strain” F059 was 100% identical to the previously reported HMD strain [19] and most closely related to ruminant *Bartonella* species [Table 3]. For the *rpoB* sequences, DNA of “strain” F059 was also 100% identical to strain HMD and with the closest related species being *B. bovis* and other ruminant *Bartonella* species [Table 3]

**Table 2:** Number of bacteremic and/or seropositive dogs, jackals, and foxes for *Bartonella* species in Iraq.

	No. (%) PCR positive*		No. (%) IFA positive †						
Canid species (no. tested)	*Candidatus* Bartonella merieuxii	*B. vinsonii* subsp. *berkhoffii*	*Bartonella henselae*	*Bartonella clarridgeiae*	*B. vinsonii* subsp. *berkhoffii*	*Bartonella bovis*	Any *Bartonella* spp.	No. (%) PCR positive & IFA positive	No. (%) PCR positive & IFA negative
*Canis familiaris* (97)	36 (37)	0	43 (44)	38 (39)	37 (38)	44 (45)	46 (47)	33 (34)	3 (3)
*Canis aureus* (57)	7 (12)	1 (2)	20 (35)	21 (37)	19 (33)	20 (35)	23 (40)	6 (11)	1 (2)
*Vulpes vulpes* (39)	0	0	2 (5)	1 (3)	2 (5)	5 (13)	5 (13)	0	0

* A positive polymerase chain reaction test result denotes a bacteremic individual.

† An indirect immunofluorescent antibody assay denotes a seropositive individual.

**Table 3:** Similarity between dog strain F059 and closest *Bartonella* species for *glt*A, ITS and *rpo*B*.

*Bartonella* species	Base-pair similarity(%) with *glt*A	Base-pair similarity(%) with ITS	Base-pair similarity(%) with *rpo*B
HMD strain	278/278 (100)JX073031	420/420 (100)EF614393	593/593 (100)EF592104
*B. bovis*	259/278 (93)AF293394	218/248 (88)AY116638	738/776 (95)AY166581
*B. schoenbuchensis*	261/278 (94)AJ278183	289/314 (92)AY116639	724/774 (94)AY167409
*B. melophagi*	261/278 (94)AY724769	243/277 (88)JF834886	725/776 (93)EF605288
*B. chomelii*	260/278 (94)AY254308	286/314 (91)AB498010	723/776 (93)AB290189
*B. capreoli*	258/278 (93)AF293392	270/302 (89)AB498009	722/774 (93)AB290188

* Percentage and GenBank accession numbers.

**Figure 1:**
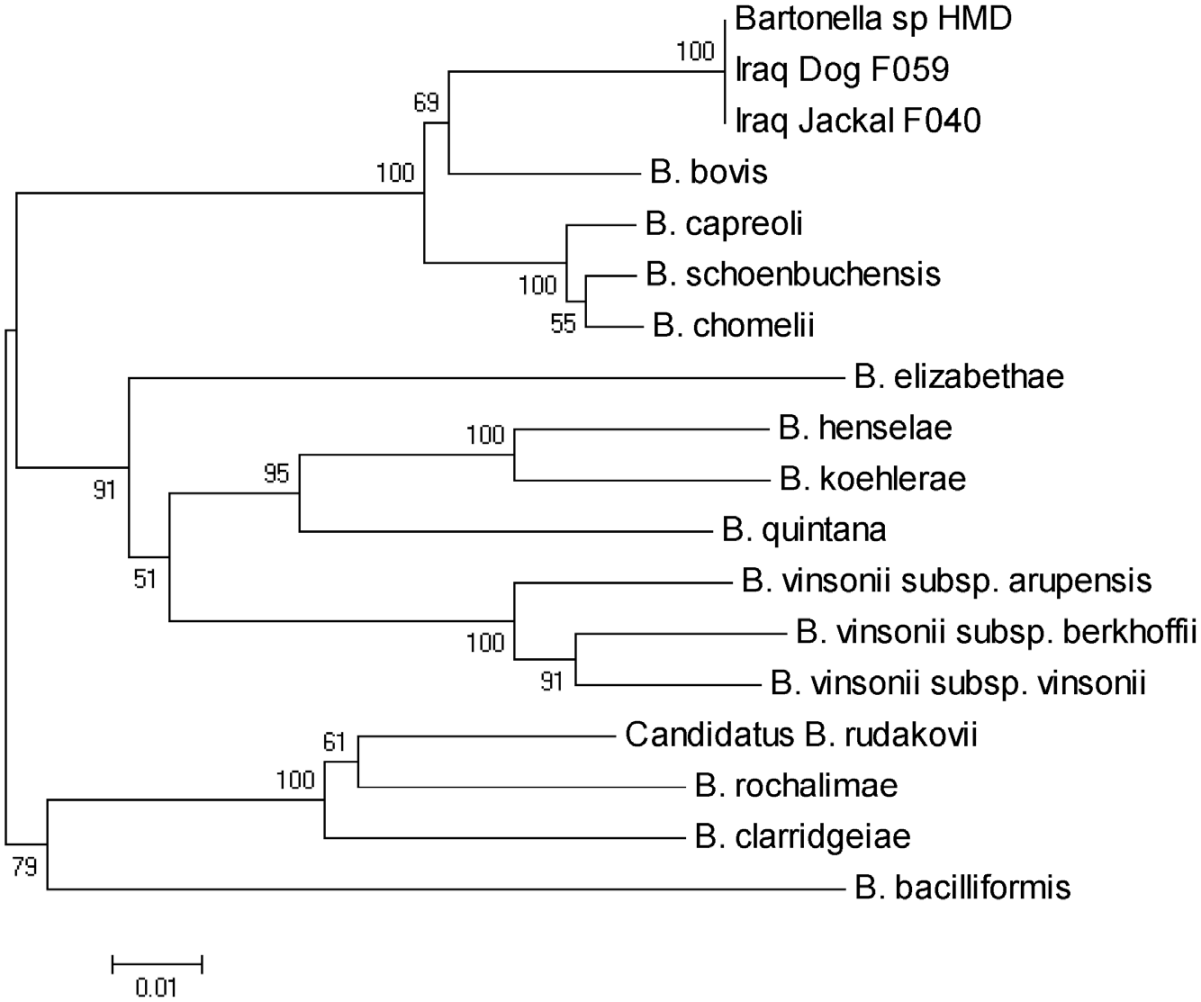
Phylogenetic tree of *Candidatus* B. merieuxii and other *Bartonella* species. The phylogenetic tree was based on concatenated sequences of two housekeeping genes (gltA and rpoB) and the 16S-23S rRNA intergenic spacer region and was constructed from 941 base-pair sequences, inferred using the Neighbor-Joining method, and 1000 replicates in the bootstrap test. Scale bar indicates 10 substitutions per nucleotide position.

A phylogenetic tree of the concatenated sequences of the *gltA* and *rpoB* genes and the 16S-23S ITS indicted that all strains clustered together and aligned most closely to *B. bovis* (Figure 1). However, partial sequences were less than 95% similar for all genes, suggesting that these canid strains likely belong to a new *Bartonella* species. DNA of other *Bartonella* species, including *B. vinsonii* subsp. *berkhoffii* was not PCR amplified from the blood of these dogs. The 36 PCR positive dogs were from either Baghdad (n = 30, 39%) or North Iraq (n = 6, 40%). Within Baghdad and its suburbs, 57.14% (20/35) of the dogs from Inner Baghdad, 40% (6/15) from Outer Baghdad and 14.8% (4/27) from North Baghdad were PCR positive. Of the male dogs, 46.5% (20/43) were PCR positive compared to 46.8% (15/32) of the females. Only 10% (1/10) of the young dogs compared to 53.3% (32/60) of the adults were PCR positive.

Overall, 47.4% of the dogs (46/97; 95% CI: 37.5%–57.4%) were seropositive to at least one *Bartonella* antigen [Table 2]. Among the 97 dogs, 34% (33/97) were both PCR and serology positive, 3.1% (3/97) were PCR positive and seronegative, 13.4% (13/97) were seropositive and PCR negative, and 49.5% (48/97) were both PCR and seronegative [Table 2]. Of the 36 PCR positive dogs, 66.6% (24/36) were seropositive for all four antigens and five dogs were seropositive for three antigens, with 86.1% (31/36) being seropositive for *B. bovis*, specifically.

### Jackals

Of the 57 jackals sampled, 55.6% (30/54) were collected from North Baghdad, 18.5% (10/54) from Outer Baghdad, 16.7% (9/54) from North Iraq, and 9.3% (5/54) from West Iraq (complete location data was missing for three jackals). The sex ratio of the sampled jackals was 83% (44/53) male and 17% (9/53) female (sex data was absent for four jackals). Age was recorded dichotomously for 51 jackals, with 74.5% (38/51) classified as adults and 25.5% (13/51) as young. Seven jackals (12.3%; 95% CI: 5.08%–23.68%) were PCR positive for *Bartonella*, including one young male from North Iraq infected with *B. vinsonii* subsp. *berkhoffii* [Table 2]. The other six jackals infected with a “strain” identical to strain HMD were also male, four being from North Baghdad and two from North Iraq. Five of these animals were reported as adults and age classification was missing from one sample.

DNA of a representative jackal “strain” (F040) was chosen for genetic analysis. The *gltA* gene sequence aligned most closely with *B. bovis* and the other ruminant *Bartonella* species [Table 4]. The 16S-23S ITS region sequence of DNA from jackal “strain” F040 was 100% identical to the HMD strain, aligning most closely with *B. schoenbuchensis* and the other ruminant *Bartonella* species. For the *rpoB* sequences, DNA amplification from jackal F040 was 100% identical to strain HMD and aligned most closely with *B. bovis* and the other ruminant *Bartonella* species [Table 4].

**Table 4:** Similarity between jackal strain F040 and closest *Bartonella* species for *glt*A, ITS and rpoB*.

*Bartonella* species	Base-pair similarity(%) with *glt*A	Base-pair similarity(%) with ITS	Base-pair similarity(%) with *rpo*B
HMD strain	278/278 (100)JX073031	426/426 (100)FJ177635	593/593 (100)EF921104
*B. bovis*	262/278 (94)AF293394	225/254 (89)AY116638	736/774 (95)AY166581
*B. schoenbuchensis*	259/278 (93)AJ278183	296/320 (93)AY116639	722/772 (94)AY167409
*B. melophagi*	261/278 (94)AY724769	250/283 (88)JF834886	723/774 (93)EF605288
*B. chomelii*	260/278 (94)AY254308	293/320 (92)AB498010	721/774 (93)AB290189
*B. capreoli*	258/278 (93)AF293392	277/308 (90)AB498009	720/772 (93)AB290188

* Percentage and GenBank accession numbers.

Overall, 40.4% (23/57; 95% CI: 27.6%–53.1%) of the jackals were seropositive for at least one *Bartonella* antigen [Table 2]. Of the seven PCR positive jackals, only one was seronegative, which was a *Bartonella* sp. strain HMD adult male from North Baghdad. All six other jackals were IFA positive for the four *Bartonella* antigens. Of the 17 seropositive jackals that were PCR negative, 70.6% (12/17) were seropositive for the four antigens tested.

### Foxes

Blood samples were collected from 39 red foxes, comprised of 21 from West Iraq, 15 from Baghdad (four from Inner and 11 from Outer Baghdad) and three from North Iraq. There were 20 male and 19 female foxes, including 29 adults and 10 young individuals. None of the fox samples were *Bartonella* PCR positive [Table 2]. Seroprevalence in foxes was 12.8% (5/39; 95% CI: 4.3%–27.4%).

### Comparisons among canids

Species, sex, age and location data were collected for most of the canids and submitted to a univariate analysis [Tables 5 and 6). Species and age were then included in the multivariate models for both PCR and serology outcomes [Tables 7 and 8]. Adjusting for age, the odds of dogs being *Bartonella* PCR positive were 11.94 times higher than of wild canids (95% CI: 4.55–31.35). Adjusting for species, the odds of adult canids being PCR positive were 5.62 times higher than young canids (95% CI: 1.19–26.59). The age-adjusted odds of dogs being *Bartonella* seropositive were 8.71 times higher than for foxes (95% CI: 3.03–25.01). Similarly, age-adjusted odds of jackals being *Bartonella* seropositive were 5.64 times higher than of foxes (95% CI: 1.87–17.07).

**Table 5:** Univariate analysis* of factors associated with *Bartonella* PCR detection in Iraqi canids.

Variable	Category	No. (%) PCR positive	Odds ratio	95% CI	p-value
*SPECIES*	Wild canid	7 (7)	Reference		
	Dog	36 (37)	7.5	3.14–17.96	<0.001
*SEX*	Female	15 (25)	Reference		
	Male	27 (25)	1.04	0.50–2.14	0.926
*AGE*	Young	2 (6)	Reference		
	Adult	37 (29)	6.37	1.45–28.0	0.006
*LOCATION*	Outside Baghdad	9 (15)	Reference		
	Baghdad	34 (26)	2.01	0.89–4.50	0.088

* Via logistic regression, with proportion PCR positive, odds ratios, 95% confidence intervals, and chi-square p-values.

**Table 6:** Univariate analysis* of factors associated with *Bartonella* serostatus in Iraqi canids.

Variable	Category	No. (%) seropositive	Odds ratio	95% CI	p-value
*SPECIES*	Fox	5 (13)	Reference		
	Jackal	23 (40)	4.60	1.57–13.51	0.006
	Dog	46 (47)	6.13	2.21–17.01	<0.001
*SEX*	Female	24 (39)	Reference		
	Male	46 (43)	1.16	0.61–2.21	0.645
*AGE*	Young	10 (30)	Reference		
	Adult	56 (44)	1.81	0.80–4.12	0.155
*LOCATION*	West Iraq	5 (17)	Reference		
	North Iraq	13 (48)	4.46	1.31–15.16	0.068
	South Iraq	1 (50)	4.80	0.26–90.29	0.295
	Inner Baghdad	23 (59)	6.90	2.17–21.91	0.001
	North Baghdad	21 (37)	2.80	0.93–8.44	0.017
	Outer Baghdad	10 (28)	1.85	0.55–6.18	0.320

* Via logistic regression, with proportion PCR positive, odds ratios, 95% confidence intervals, and chi-square p-values. An individual is considered *Bartonella* seropositive if IFA test positive for at least one of *B.henselae*, *B. clarridgeiae*, *B. vinsonii* subsp. *berkhoffii*, or *B. bovis*.

**Table 7:** Multivariate analysis* of factors associated with *Bartonella* PCR detection in Iraqi canids.

Variable	Category	Coefficient estimate	Odds ratio	95% CI	p-value
*SPECIES*	Wild canid	Reference			
	Dog	2.48	11.94	4.55–31.35	<0.001
*AGE*	Young	Reference			
	Adult	1.73	5.62	1.19–26.59	0.029

* Via logistic regression, with coefficients, odds ratios, 95% confidence intervals, and chi-square p-values for variables included in the main effects model.

**Table 8:** Multivariate analysis* of factors associated with *Bartonella* serostatus in Iraqi canids.

Variable	Category	Coefficient estimate	Odds ratio	95% CI	p-value
*SPECIES*	Fox	Reference			
	Jackal	1.73	5.64	1.87–17.07	0.002
	Dog	2.16	8.71	3.03–25.01	<0.001
*AGE*	Young	Reference			
	Adult	0.55	1.73	0.72–4.16	0.224

* Via logistic regression, with coefficients, odds ratios, 95% confidence intervals, and chi-square p-values for variables included in the main effects model. An individual is considered *Bartonella* seropositive if IFA test positive for at least one of *B.henselae*, *B. clarridgeiae*, *B. vinsonii* subsp. *berkhoffii*, or *B. bovis*.

## Discussion

This study reports on the prevalence of *Bartonella* species in domestic and wild canids of Iraq and provides the first detection of *Bartonella* in jackals. Dogs and jackals were frequently infected with a new *Bartonella* species similar to strain HMD, initially described from dogs in southern Italy and Greece [19] and more recently in two dogs from Sri Lanka [32]. The PCR prevalence in Iraqi dogs (45.4%) was similar to the prevalence in the dogs from Basilicata, Italy (42.8%, 6/14).

Based on the criteria suggested by La Scola et al. [33], the low similarity percentages (<95.5%) between the Iraqi, Greek, Italian and Sri Lankan “strains” and *B. bovis* clearly suggest that the “strains” infecting dogs belong to a new *Bartonella* species. We propose *Candidatus* Bartonella merieuxii for this new species. *B. vinsonii* subsp. *berkhoffii*, a common *Bartonella* species isolated from dogs [3], was not detected in the Iraqi dogs and detected only from one jackal. Similarly, *B. rochalimae* was not detected from any of the canids, which is especially interesting as red foxes have been found to be infected by this *Bartonella* species in Israel and Europe [30]. Given the statistically significantly higher PCR prevalence of *Candidatus* B. merieuxii infection in dogs than in wild canids, domestic dogs may represent a major reservoir of this new species in Iraq. Because this new species is genetically related to *B. bovis*, we sought to investigate the seroprevalence of *B. bovis* in these canids in addition to the antigens usually used for canids in our laboratory. Most (86%) dogs and jackals that were PCR positive were also seropositive for *B. bovis* and for the three other antigens. The lowest seroprevalence observed was for *B. clarridgeiae* and *B. vinsonii* subsp. *berkhoffii*, suggesting that these *Bartonella* species may be found less frequently in Iraqi canids as compared to other parts of the world.

The high seroprevalence in stray dogs and jackals correlates with previously reported studies on seroprevalence of *Bartonella* species in animals of the tropics, Middle East, and some African nations [1], [2]. A recent study of stray dogs in the tropics [32] reported an overall seroprevalence of 8.3% (41/455), ranging from 0% in dogs from Vietnam to almost 11% in dogs from Bogota, Colombia. These data contrast with reports in domestic pet dogs in the United States and Europe, where overall seroprevalence is <5% [1]. In Thailand, prevalence of *Bartonella* bacteremia in stray dogs was 31.3% (60/192) [17].

Cross-reactivity is known to occur between *B. henselae* and *B. clarridgeiae*, especially in human sera [2], and therefore it is unclear whether the seropositivity for more than one *Bartonella* species was due to cross-reactions or to co-infection or sequential infection to different *Bartonella* spp. over an extended time period. Given the relatively small sample sizes of some canid groups and locations, larger studies will be important to strengthen our findings.

To date, no vector has been proven as the source of infection for any *Bartonella* spp. in dogs. The current hypothesis on the mode of transmission of *B. vinsonii* subsp. *berkhoffii* involves ticks, because seropositive dogs were also frequently seropositive for other known tick-borne pathogens [3], [34], [35]. Similarly, although the body louse is considered the primary vector for *B. quintana*, *B. quintana* DNA has been amplified from both fleas and ticks, providing support for being possible *Bartonella* vectors [9], [36]. Furthermore, *Candidatus* Bartonella merieuxii was detected in *Rhipicephalus sanguineus* ticks, as DNA was amplified from three tick pools including one pool from salivary glands from female ticks and one gut content pool collected two dogs PCR positive for *Candidatus* B. merieuxii [19]. Therefore, ticks may be possible candidate vectors for this new *Bartonella* species. Unfortunately, information on the presence of ectoparasites was not available and no quantitative measure of the fleas and ticks was reported. Given the high prevalence of *Bartonella* spp. reported in dogs and to a lesser extent in jackals, studies to detect *Bartonella* spp. DNA in arthropods in Iraq are warranted.

In humans, clinical diagnosis of atypical CSD and bacteremia associated with other emerging clinical manifestations of bartonellosis is difficult [2], [3]. Domestic cats are generally asymptomatic carriers of *B. henselae* and *B. clarridgeiae*. Symptomatic bacteremic dogs are often seronegative and frequently manifest similar pathology and disease manifestations as reported in human patients [3], [19], [37]. Furthermore, most of the *Bartonella* species identified in sick dogs are also pathogenic for humans [2]. *Bartonella quintana*, for instance, causes a high percentage of endocarditis in people and has been associated with endocarditis in dogs [2], [38], [39]. Therefore, seroprevalence in Iraqi dog owners and bacteremia in Iraqi people with unexplained fever or culture negative endocarditis requires further investigation. This is especially true in Baghdad and the surrounding region, where most of the positive dogs were detected. Similarly, it will be important to investigate pre and post-deployment seroprevalence in United States military personnel who were stationed in Iraq. It would be critical to test for *Bartonella* bacteremia in veterans with unexplained fever, lymphadenopathy, or culture negative endocarditis. Finally, it will also be essential to test any dog brought back from Iraq to the USA for presence of *Bartonella* bacteremia to prevent any accidental introduction of a new *Bartonella* species to the New World.

In the context of zoontic bartonellosis, the dog appears to serve as both a sentinel and a reservoir for human infections. As a sentinel, infection in both dogs and humans (most often cases of endocarditis) has now been documented for eight *Bartonella* species or subspecies. As a reservoir, persistent infection in dogs has been documented in association with *B. vinsonii* subsp. *berkhoffii*, *B. henselae* and *B. koehlerae* [3]. As such, there are reports of dog bite transmission of *B. henselae* to people [40] and two recent publications that implicate needle stick transmission *B. vinsonii* subsp. *berkhoffii or B. henselae* from dogs to veterinarians [41], [42]. In the context of diagnostic testing, several of our laboratories are attempting isolation of this novel *Bartonella* sp. to facilitate additional genetic characterization and the development and validation of *Bartonella* species specific serologic assays. In addition, attempts at isolation from sick soldiers deployed in Iraq would seem reasonable.

In summary, presence of *Bartonella* infection was detected in domestic dogs and wild canids in different areas of Iraq. Our findings identify a potential role for dogs as a major reservoir of a new and potentially zoonotic *Bartonella* species.

### Description of *Candidatus* Bartonella merieuxii sp. nov

*Bartonella merieuxii* (méri.eu.xii N. L. gen. N. *merieuxii* of Mérieux, in honor of Charles Mérieux, a French physician, founder of the Mérieux Foundation and former C.E.O. of the Mérieux Institute in Lyon, France. His broad interest in comparative medicine and in one health, within the Pasteur's tradition at its best (“No borders between the two medicines” was his favorite saying), allowed him to build many bridges between physicians and veterinarians in the field of zoonoses. DNA of this species has been amplified and sequenced from dogs and wild canids (jackals) in the old world.

## References

[1] Boulouis HJ, Chang CC, Henn JB, Kasten RW, Chomel BB (2005) Factors associated with the rapid emergence of zoonotic *Bartonella* infections. Vet Res 36: 383–410. 15845231 10.1051/vetres:2005009

[2] Chomel BB, Boulouis HJ, Maruyama S, Breitschwerdt EB (2006) *Bartonella* spp. in pets and effect on human health. Emerg Infect Dis 12: 389–94. 16704774 10.3201/eid1203.050931 PMC3291446

[3] Breitschwerdt EB, Maggi RG, Chomel BB, Lappin MR (2010) Bartonellosis: an emerging infectious disease of zoonotic importance to animals and human beings. J Vet Emerg Crit Care (San Antonio) 20: 8–30. 20230432 10.1111/j.1476-4431.2009.00496.x

[4] Dehio C (2001) *Bartonella* interactions with endothelial cells and erythrocytes. Trends Microbiol 9 6:279–85. 11390243 10.1016/s0966-842x(01)02047-9

[5] Duncan AW, Maggi RG, Breitschwerdt EB (2007) A combined approach for the enhanced detection and isolation of *Bartonella* species in dog blood samples: pre-enrichment liquid culture followed by PCR and subculture onto agar plates. J Microbiol Methods 69: 273–278. 17346836 10.1016/j.mimet.2007.01.010

[6] Gundi VA, Bourry O, Davoust B, Raoult D, La Scola B (2004) *Bartonella clarridgeiae* and *B. henselae* in dogs, Gabon. Emerg Infect Dis 10: 2261–2262. 15672535 10.3201/eid1012.040359 PMC3323364

[7] Bown KJ, Bennet M, Begon M (2004) Flea-borne *Bartonella grahamii* and *Bartonella taylorii* in bank voles. Emerg Infect Dis 10: 684–687. 15200860 10.3201/eid1004.030455 PMC3323072

[8] Morick D, Krasnov BR, Khokhlova IS, Gottlieb Y, Harrus S (2011) Investigation of *Bartonella* acquisition and transmission in *Xenopsylla ramesis* fleas (Siphonaptera: Pulicidae). Mol Ecol 20: 2864–2870. 21692752 10.1111/j.1365-294X.2011.05033.x

[9] Chang CC, Chomel BB, Kasten RW, Romano V, Tietze N (2001) Molecular evidence of *Bartonella* spp. in questing adult *Ixodes pacificus* ticks in California. J Clin Microbiol 39: 1221–1226. 11283031 10.1128/JCM.39.4.1221-1226.2001 PMC87914

[10] Halos L, Jamal T, Maillard R, Girard B, Guillot J, et al (2004) Role of Hippoboscidae flies as potential vectors of *Bartonella* spp. infecting wild and domestic ruminants. Appl Environ Microbiol 70: 6302–6305. 15466580 10.1128/AEM.70.10.6302-6305.2004 PMC522062

[11] Reis C, Cote M, Le Rhun D, Lecuelle B, Levin ML, et al (2011) Vector competence of the tick *Ixodes ricinus* for transmission of *Bartonella birtlesii* . PLoS Negl Trop Dis 5 5:e1186. 21655306 10.1371/journal.pntd.0001186 PMC3104967

[12] Chomel BB, Kasten RW, Sykes JE, Boulouis HJ, Breitschwerdt EB (2003) Clinical impact of persistent *Bartonella* bacteremia in humans and animals. Ann N Y Acad Sci 990: 267–278. 12860639 10.1111/j.1749-6632.2003.tb07376.x

[13] Brouqui P, Raoult D (2006) New insight into the diagnosis of fastidious bacterial endocarditis. FEMS Immunol Med Microbiol 47: 1–13. 16706783 10.1111/j.1574-695X.2006.00054.x

[14] Chomel BB, Kasten RW, Williams C, Wey AC, Henn JB, et al (2009) *Bartonella* endocarditis: a pathology shared by animal reservoirs and patients. Ann N Y Acad Sci 1166: 120–126. 19538271 10.1111/j.1749-6632.2009.04523.x

[15] Jacobs RF, Schutze GE (1998) *Bartonella henselae* as a cause of prolonged fever and fever of unknown origin in children. Clin Infect Dis 26: 80–84. 9455513 10.1086/516256

[16] Maggi RG, Mascarelli PE, Pultorak EL, Hegarty BC, Bradley JM, et al (2011) *Bartonella* spp. bacteremia in high-risk immunocompetent patients. Diagn Microbiol Infect Dis 71: 430–437. 21996096 10.1016/j.diagmicrobio.2011.09.001

[17] Bai Y, Kosoy MY, Boonmar S, Sawatwong P, Sangmaneedet S, et al (2010) Enrichment culture and molecular identification of diverse *Bartonella* species in stray dogs. Vet Microbiol 146: 314–319. 20570065 10.1016/j.vetmic.2010.05.017

[18] Henn JB, Gabriel MW, Kasten RW, Brown RN, Koehler JE, et al (2009) Infective endocarditis in a dog and the phylogenetic relationship of the associated “*Bartonella rochalimae*” strain with isolates from dogs, gray foxes, and a human. J Clin Microbiol 47: 787–790. 19109472 10.1128/JCM.01351-08 PMC2650912

[19] Diniz PP, Billeter SA, Otranto D, De Caprariis D, Petanides T, et al (2009) Molecular documentation of *Bartonella* infection in dogs in Greece and Italy. J Clin Microbiol 47: 1565–1567. 19261798 10.1128/JCM.00082-09 PMC2681825

[20] Pérez C, Maggi RG, Diniz PP, Breitschwerdt EB (2011) Molecular and serological diagnosis of *Bartonella* infection in 61 dogs from the United States. J Vet Intern Med 25: 805–810. 21615498 10.1111/j.1939-1676.2011.0736.x

[21] Henn JB, Gabriel MW, Kasten RW, Brown RN, Theis JH, et al (2007) Gray foxes (*Urocyon cinereoargenteus*) as a potential reservoir of a *Bartonella clarridgeiae*-like bacterium and domestic dogs as part of a sentinel system for surveillance of zoonotic arthropod-borne pathogens in northern California. J ClinMicrobiol 45: 2411–2418. 10.1128/JCM.02539-06 PMC1951249 17553970

[22] Havas KA, Burkman K (2011) A comparison of the serological evidence of *Coxiella burnetii* exposure between military working dogs and feral canines in Iraq. Mil Med 176: 1101–1103. 22128642 10.7205/milmed-d-11-00025

[23] Chomel BB, Abbott C, Kasten RW, Floyd-Hawkins KA, Kass PH, et al (1995) *Bartonella henselae* prevalence in domestic cats in California: Risk factors and association between bacteremia and antibody titers. J Clin Microbiol 33: 2445–2450. 7494043 10.1128/jcm.33.9.2445-2450.1995 PMC228433

[24] Chomel BB, Carlos ET, Kasten RW, Yamamoto K, Chang CC (1999) *Bartonella henselae* and *Bartonella clarridgeiae* infection in domestic cats from The Philippines. Am J Trop Med Hyg 60: 593–597. 10348234 10.4269/ajtmh.1999.60.593

[25] Chang CC, Kasten RW, Chomel BB, Simpson DC, Hew CM, et al (2000) Coyotes (*Canis latrans*) as the reservoir for a human pathogenic *Bartonella* sp.: molecular epidemiology of *Bartonella vinsonii* subsp. *berkhoffii* infection in coyotes from central coastal California. J Clin Microbiol 38: 4193–4200. 11060089 10.1128/jcm.38.11.4193-4200.2000 PMC87562

[26] Norman AF, Regnery R, Jameson P, Greene C, Krause DC (1995) Differentiation of *Bartonella*-like isolates at the species level by PCR-restriction fragment length polymorphism in the citrate synthase gene. J Clin Microbiol 33: 1797–1803. 7545181 10.1128/jcm.33.7.1797-1803.1995 PMC228273

[27] Jensen WA, Fall MZ, Rooney J, Kordick DL, Breitschwerdt EB (2000) Rapid identification and differentiation of *Bartonella* species using a single-step PCR assay. J Clin Microbiol 38: 1717–1722. 10790087 10.1128/jcm.38.5.1717-1722.2000 PMC86570

[28] Renesto P, Gouvernet J, Drancourt M, Roux V, Raoult D (2001) Use of *rpoB* gene analysis for detection and identification of *Bartonella* species. J Clin Microbiol 39: 430–437. 11158086 10.1128/JCM.39.2.430-437.2001 PMC87755

[29] Thompson JD, Higgins DG, Gibson TG (1994) CLUSTAL W: improving the sensitivity of progressive multiple sequence alignment through sequence weighting, position-specific gap penalties and weight matrix choice. Nucleic Acids res 22: 4673–4674. 7984417 10.1093/nar/22.22.4673 PMC308517

[30] Henn JB, Chomel BB, Boulouis HJ, Kasten RW, Murray WJ, et al (2009) *Bartonella rochalimae* in raccoons, coyotes, and red foxes. Emerg Infect Dis 15: 1984–1987. 19961681 10.3201/eid1512.081692 PMC3044513

[31] Tamura K, Peterson D, Peterson N, Stecher G, Nei M, et al (2011) MEGA5: molecular evolutionary genetics analysis using maximum likelihood, evolutionary distance, and maximum parsimony methods. Mol Biol Evol 28: 2731–2739. 21546353 10.1093/molbev/msr121 PMC3203626

[32] Brenner EC, Chomel BB, Singhasivanon OU, Namekata DY, Kasten RW, et al. (2012) *Bartonella* infection in urban and rural dogs from the tropics: Brazil, Colombia, Sri Lanka and Vietnam. Epidemiol Infect 140 [ahead of print] doi:10.1017/S0950268812000519. 10.1017/S0950268812000519 PMC9152078 22459880

[33] La Scola B, Zeaiter Z, Khamis A, Raoult D (2003) Gene-sequence-based criteria for species definition in bacteriology: the Bartonella paradigm. Trends Microbiol 11: 318–321. 12875815 10.1016/s0966-842x(03)00143-4

[34] Billeter SA, Levy MG, Chomel BB, Breitschwerdt EB (2008) Vector transmission of *Bartonella* species with emphasis on the potential for tick transmission. Med Vet Entomol 22: 1–15. 18380649 10.1111/j.1365-2915.2008.00713.x

[35] Angelakis E, Billeter SA, Breitschwerdt EB, Chomel BB, Raoult D (2010) Potential for tick-borne bartonelloses. Emerg Infect Dis 16: 385–391. 20202411 10.3201/eid1603.091685 PMC3322042

[36] Rolain JM, Franc M, Davoust B, Raoult D (2003) Molecular detection of *Bartonella quintana*, *B. koehlerae*, *B. henselae*, *B. clarridgeiae*, *Rickettsia felis*, and *Wolbachia pipientis* in cat fleas, France. Emerg Infect Dis 9: 338–342. 12643829 10.3201/eid0903.020278 PMC2958535

[37] Honadel TE, Chomel BB, Yamamoto K, Chang C, Farver TB (2001) Seroepidemiology of *Bartonella vinsonii* subsp. *berkhoffii* exposure among healthy dogs. J Am Vet Med Assoc 219: 480–484. 11518174 10.2460/javma.2001.219.480

[38] Benslimani A, Fenollar F, Lepidi H, Raoult D (2005) Bacterial zoonoses and infective endocarditis, Algeria. Emerg Infect Dis 11: 216–224. 15752438 10.3201/eid1102.040668 PMC3320429

[39] Znazen A, Rolain JM, Hammami N, Kammoun S, Hammami A, et al (2005) High prevalence of *Bartonella quintana* endocarditis in Sfax, Tunisia. Am J Trop Med Hyg 72: 503–507. 15891120

[40] Rolain JM, Boureau-Voultoury A, Raoult D (2009) Serological evidence of *Bartonella vinsonii* lymphadenopathies in a child bitten by a dog. Clin Microbiol Infect 15 Suppl 2: 122–123. 19374641 10.1111/j.1469-0691.2008.02197.x

[41] Oliveira AM, Maggi RG, Woods CW, Breitschwerdt EB (2010) Suspected needle stick transmission of *Bartonella vinsonii* subspecies *berkhoffii* to a veterinarian. J Vet Intern Med 24 5:1229–1232. 20695992 10.1111/j.1939-1676.2010.0563.x

[42] Lin JW, Chen CM, Chang CC (2011) Unknown fever and back pain caused by *Bartonella henselae* in a veterinarian after a needle puncture: a case report and literature review. Vector Borne Zoonotic Dis 11 5:589–591. 20569013 10.1089/vbz.2009.0217

